# CD2 targeted nanoparticles containing IL-2 mimic fetal-maternal tolerance of pregnancy by inducing synergistic TGF-β-producing NK cells and Tregs

**DOI:** 10.3389/fimmu.2025.1692547

**Published:** 2025-11-24

**Authors:** David A. Horwitz, Antonio La Cava

**Affiliations:** 1 General Nanotherapeutics, Santa Monica, CA, United States; 2 Keck School of Medicine, University of Southern California, Los Angeles, CA, United States; 3 Department of Medicine, University of California, Los Angeles, Los Angeles, CA, United States; 4 Department of Medicina Molecolare e Biotecnologie Mediche, Federico II University of Naples, Naples, Italy

**Keywords:** maternal-fetal tolerance, T regulatory cells (Tregs), natural killer (NK) cells, transforming growth factor β (TGF-β), nanoparticles

## Abstract

T regulatory cells (Tregs) are essential for maintaining immune homeostasis and tolerance. We have reported that CD2-targeted nanoparticles containing IL-2 induce CD4 and CD8 Foxp3^+^ Tregs, together with TGF-β-producing CD56^bright^ NK cells. Generation and maintenance of stable Tregs critically depended on TGF-β dependent interactions between these adaptive and innate immune cells. This resembles what occurs in maternal-fetal tolerance in pregnancy, where TGF-β from uterine stromal cells and fetal trophoblasts induces NK cells and T cells to become decidual Tregs and NK cells that prevent fetal rejection. Thus, both in the periphery and *in utero*, a TGF-dependent crosstalk between NK cells and Tregs appears vital for maintaining immune tolerance. This mimicry of maternal-fetal tolerance has clinical relevance. Subjects with SLE and other autoimmune disorders have deficits in IL-2, TGF-β and NK cells. It is likely that recent clinical trials in these diseases with IL-2 that did not reach their primary end points because they failed to address these additional defects. Our anti-CD2 conjugated NPs target CD2-bearing T cells and NK cells and provide them with IL-2 and TGF-β *in vivo* in order to repair both cytokine defects. Moreover, the NPs do not need to be encapsulated with TGF-β because the NK cell-derived TGF-β produced locally by the target cells is sufficient to sustain Tregs. For these reasons, we believe this NP platform has great potential not only for the prevention and/or treatment of a wide variety of immune-mediated disorders, but also to prevent recurrent miscarriages.

## Introduction

The pleotropic cytokine transforming growth factor β (TGF-β) has a critical role in the generation, function, and maintenance of T regulatory cells (Tregs) (1, 2). We recently reported that anti-CD2-decorated nanoparticles (NPs) containing IL-2 prevent a fatal graft versus host disease (GVHD in humanized mice (3). In this model, the tolerogenic effects of IL-2 on the generation of CD4 and CD8 Foxp3^+^ Tregs were TGF-β-dependent. Blocking TGF-β signaling converted the tolerogenic effects of our NPs to pathologic immunogenic effects that accelerated the demise of the mice (4). Importantly, the NPs also induced a subset of NK cells that produced TGF-β that stabilized newly induced Tregs, endowing them with long-lasting functional stability. Both Treg generation and maintenance depended on a synergistic crosstalk between Tregs and TGF-β-producing NK cells (5). Thus, the NPs induced both Treg and TGF-β producing NK cells and this TGF-β helped stabilize new regulatory T cells and maintain their lasting function.

In pregnancy, TGF-β has an essential role not only for hormone secretion, placental development and embryonic growth, but also in preventing rejection of the semi-allogenic fetus bearing paternal antigens (6). Notably, in pregnancy, interactions between TGF-β dependent Tregs and NK cells in the decidua support each other to prevent fetal rejection (7).

**Figure 1:**
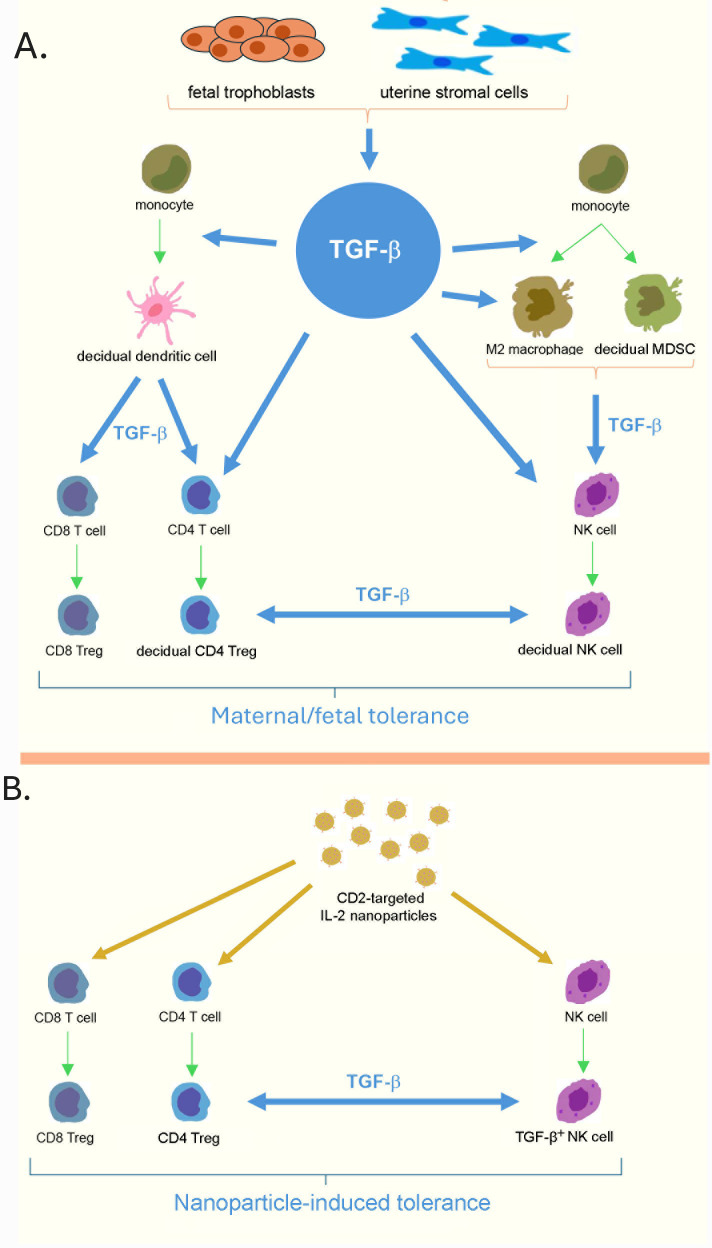
The critical role of TGF-β in fetal maternal tolerance. **(A)** TGF-β produced by fetal trophoblasts and uterine stromal cells induces monocytes to differentiate into tolerogenic decidual dendritic cells (dDC), M2 macrophages, and decidual myeloid-derived suppressor cells (MDSC). All these cell types produce TGF-β. TGF-β promotes the differentiation of CD4 cells into CD4 Tregs and supports the development of suppressive functions in CD8 cells. It also drives migrating NK cells to become specialized decidual NK cells (dNK cells). Both dNK cells and dTregs generate TGF-β, and their combined actions are essential for maternal-fetal tolerance. **(B)** The effects of CD2 targeted NPs on peripheral T cells and NK cells that lead to CD4 and CD8 Tregs and the TGF-β producing NK cells involved in the generation, stabilization and maintenance of the Tregs.

Based on these considerations, we propose that our TGF-β-inducing nanoparticles may mimic the physiological function of the maternal-fetal tolerance. In both there is an interaction between NK cells and Tregs as shown in **Figure 1**. While in pregnancy this ensures a robust maternal-fetal tolerance, with our NPs this interaction offers a new immunotherapeutic approach to suppress pro-inflammatory responses. Here we discuss the similarities between the Tregs and NK cells induced *in vivo* by our NPs and the Tregs and NK cells involved in maternal-fetal maternal tolerance, together with the key role of TGF-β on their activities.

## TGF-β and decidual NK cells

Innate NK cells represent the first line of defense against viruses and tumors and are schematically divided into two major subsets: a predominant CD56^dim^CD16^+^ subset with cytotoxic properties, and a smaller CD56^bright^CD16^-^ subset that can produce immunogenic (IFN-γ) or tolerogenic (TGF-β and IL-10) cytokines (8).

In early pregnancy, endometrial stromal cells and trophoblasts produce chemokines that recruit NK cells to the fetal-maternal interface (6). Progesterone released from the corpus luteum upregulates the production of TGF-β by these cells, converting CD56^dim^CD16^+^ NK cells into decidual CD56^bright^CD16^-^ NK cells (9–12). Decidual NK (dNK) cells constitute 50-70% of the total number of lymphocytes in the early decidua, produce tolerogenic TGF-β and IL-10, and secrete angiogenic factors such as the VEGF which supports placental vascularization (13, 14). It is noteworthy that these NK cells lack the killer immunoglobulin receptors (KIRs) typical of NK cells, while they express the inhibitory receptor complex NKG2A/CD94 that binds to HLA-E molecules on the extravillous trophoblasts (13). Trophoblast expression of HLA-G and NK cell ligation of KIR2DL4 are upregulated by TGF-β, resulting in decreased cytotoxic activity (13, 15). Moreover, at the maternal-fetal interface, dNK cells suppress Th17-mediated inflammation (16), with the dCXCR4^+^ CD56^bright^CD16^-^ NK cell subset playing an important role in maternal-fetal tolerance (17). Thus, in pregnancy the TGF-β is derived from the effects of progesterone on decidual trophoblasts and stromal cells whereas our NPs induce the NK cells induced to produce this cytokine.

## TGF-β and decidual Tregs

In addition to NK cells, the TGF-β produced by stromal cells and trophoblasts also plays a major role in the generation of tissue-specific decidual (d)Tregs (7). Interestingly, both thymic-derived (t)Tregs and newly induced peripheral (p)Tregs can differentiate into dTregs. The TGF-β stabilizes and maintains the dTregs in the placenta. The CD25^+^Foxp3^+^ dTregs express higher levels of co-inhibitory molecules including CTLA-4, GITR and CD39 as compared to pTregs (7), express the HLA-DR and CD69 activation markers, express CD103 (18), become CD45RO^+^ memory cells, and inhibit fetal antigen-specific T effector cells through CTLA-4-mediated suppression and the production of anti-inflammatory TGF-β. These Tregs also produce IL-10, which increases Foxp3 expression and CTLA-4-mediated immune suppression (19, 20). The critical role of CCR8^+^ dTregs in maintaining maternal-fetal immune tolerance during early pregnancy is evidenced by the ability of adoptively transferred CCR8^+^ dTregs to rescue fetal loss in abortion-prone mice (21).

Importantly, the crosstalk between dNK cells and dTregs supports the production of TGF-β by each cell subset. The TGF-β produced by dNK cells supports induction and expansion of Tregs and their maintenance long-term (14). In contrast, the production of TGF-β by dTregs inhibits the cytotoxic activity of dNK cells, thus reducing the risk of damage to the allogeneic fetal trophoblasts. TGF-β also downregulates IFN-γ production in NK cells, and reinforces self-tolerance by increasing the expression of inhibitory NK cell receptors such as NKG2A and its ligand HLA-E (22). TGF-β also facilitate placental vascular remodeling and trophoblastic invasion by promoting dNK cell secretion of the angiogenic factors VEGF and PIGF (9). Any abnormality of dTregs or dNK cells will result in an increased risk of fetal loss (6, 7, 14, 23, 24).

## TGF-β effects on other immune cells

In pregnancy, TGF-β influences multiple mononuclear immune cell populations. TGF-β induces decidual CD8^+^ cells to express PD-1, CTLA-4 and LAG3 that promote a tolerogenic state (25). In addition to the interactions between CD4^+^ T cells and NK cells, CD8^+^ T cell-NK cell interactions are also important. In both monocyte-dependent and independent T cell-dependent antibody production, NK cells play a key role in inducing CD8^+^ T cells to suppress IgG production. Specifically, we found that CD8^+^ T cells co-incubated with CD4^+^ T cells and B cells failed to inhibit IgG production. However, when NK cells were added, the CD8 cell- NK cell interaction induced NK cells to produce TGF-β which enabled CD8 cells to become suppressive cells. This TGF-β was essential since blocking TGF-β signaling abolished suppression (26).

When trophoblast’s CXCL16 ligates CXCR6 on macrophages, the TGF-β produced by these cells promotes the development of anti-inflammatory M2 macrophages and the development of tolerogenic myeloid derived suppressor cells (MDSCs) (13, 27). For the latter, crosstalk between dNK cells and MDSCs results in the induction of immunosuppressive Tregs (28). Trophoblasts also interact with dendritic cells (DCs) and the TGF-β produced by these cells promotes their tolerogenic antigen-presenting cell activity (29). These tolerogenic DCs can internalize human paternal HLA-G expressed by fetal trophoblasts and present it to T cells, inducing alloantigen-specific Tregs which prevent fetal rejection (30). Whether the TGF-β produced by the tolerogenic NK cells induced by our NPs induce also induce M2 macrophages or MDSCs is an open question.

## The role of IL-2 in maternal-fetal tolerance

IL-2 is essential for the induction and survival of Foxp3^+^ Tregs (31). Initially, this cytokine is produced by syncytiotrophoblasts (32), and subsequently by Th1 cells (32, 33). Because high levels of IL-2 cause inflammation, its production must be tightly regulated to ensure tolerogenic levels. Our CD2 targeted nanoparticles contain low dose tolerogenic levels of IL-2. Signals through the PI3K kinase pathway determine whether T cells become effector or regulatory cells. Strong signaling results in effector cells, while weak signaling results in Tregs (34). The nanomolar low dose levels of IL-2 in our NPs in the presence of TGF-β induce Tregs.

Decidual stromal cells play an important role in the regulation of IL-2. These cells upregulate IL-2R signaling (IL-2Rβ, CD122; and IL-2Rγ, CD132) to increase IL-2 uptake. Importantly, however, decidual stromal cells also decrease pSTAT5 which decreases IL-2 signaling (35). In addition, TGF-β promotes M2 macrophages and down-regulates IL-2 production (36). This effect and alleviation of fetal loss in abortion-prone mice can also be achieved by IL-2 complexed with anti-IL-2 antibodies (37).

## CD2-targeted nanoparticles containing IL-2 and CD2 interactions in pregnancy

The CD2 surface glycoprotein expressed on T cells and NK cells functions as both an adhesion molecule and a co-stimulatory receptor. It was reported years ago that combining anti-CD2 with anti-CD3 antibody treatment prolonged allograft rejection indefinitely by decreasing production of pro-inflammatory cytokines and increasing TGF-β (38). Moreover, anti-CD2 treatment can deplete memory T effector cells that reject grafts (39).

Anti-CD2 targets NK cells as well as T cells. We have previously reported that anti-CD2 treatment can induce NK cells to produce active TGF-β (40). Although both anti-CD2 and anti-CD3 treatment induced T cell proliferation, only the latter also induced IgG production because anti-CD2 treatment-induced production of TGF-β was inhibitory on antibody production. In fact, T cell-dependent B cell antibody production was rescued by TGF-β signaling blockade (41).

Turning to our CD2-targeted tolerogenic NPs, we have demonstrated disease-protective effects in lupus mice and in GvHD, where the depletion of TGF-β-producing NK cells abolished the NPs’ protective effects (5, 42).

The ligand for CD2 is CD58, also known as lymphocyte function-associated antigen 3 (LFA-3), CD58 is a key cell adhesion molecule widely expressed on antigen-presenting cells. Its primary binding partner is CD2, found on T cells and natural killer (NK) cells. While CD2-CD58 interactions are well known for T cell activation and adhesion, this interaction can also promote the generation of IL-10 secreting Tregs (43). Of particular interest, CD2-CD58 interaction is central to the formation and function of immune multicellular clusters (43). Clusters of Tregs and NK cells bound to antigen-presenting cells could promote synergistic activities in both populations.

In early pregnancy, the ligand for T cell CD2, CD58, increases (44). After ovum fertilization, the luteal cells in the *corpus luteum* expand and express high levels of CD58 (45, 46) and can produce TGF-β (47). These luteal cells produce high levels of progesterone which promote uterine stromal cells to produce TGF-β (11, 12). Stromal cells, like cDCs, also express CD58 (46), and this can result in T cells and NK cells binding to these cells. Stromal cell TGF-β production could then promote the generation of both dTregs and dNK cells (7, 43). Stromal cells’ CD58 could also promote formation of clusters of dTregs and dNK cells, with synergistic TGF-β-dependent interactions having a major role in both maternal-fetal tolerance and in placental vascularization (7) (48, 49).

It is noteworthy that our CD2-targeted NPs may replicate stromal cells’ role in generating dTregs and dNK cells that are important for maternal-fetal tolerance. PLGA NPs have a role in lymphocyte aggregation within immune microenvironments (50), and besides separately inducing Tregs and TGF-producing NK cells, they may also promote interacting aggregates that stabilize and sustain Tregs (51).

## Discussion

We have discussed the similarity in the mechanism of action of CD2 targeted NPs containing IL-2 and maternal-fetal tolerance of pregnancy. In both cases, TGF-β plays a central role. Besides targeting T cells, anti-CD2 antibodies induce NK cells to produce TGF-β (40), and the anti-CD2 antibody coating of our NPs induces a TGF-β-producing NK cell population that provides TGF-β for IL-2 induction of CD4 and CD8 Tregs *in vivo*. TGF-β is critical since blocking TGF-β signaling converted the tolerogenic effects of our NPs to immunogenic and accelerated immune-mediated inflammatory disease (5).

In pregnancy, the TGF-β produced by decidual stromal cells and trophoblasts is essential for the generation of decidual Tregs and NK cells that work together to induce the maternal-fetal tolerance that prevents rejection of the semi-allogeneic fetus. Although the analogy between our NPs and decidual cells is strong in triggering TGF-β is strong, it is not identical since the first is exogenous and the second is endogenous.

When anti-CD2 targeted NPs containing IL-2 are cultured *in vitro* with PBMC, cell clusters appear within 24 hours. Studying the cell populations in these clusters using immunofluorescence or confocal microscopy can help identify NK cells and newly induced Tregs related to TGF-β secretion, supporting that our NPs function as tolerogenic artificial antigen-presenting cells (3).

We want to reiterate that the central role of TGF-β in the tolerogenic effects of IL-2 on Tregs has potential clinical significance. Due to the involvement of IL-2 in the development and stabilization of CD4^+^ FOXP3^+^ pTregs (52–54), there is ongoing interest in using IL-2 or IL-2 muteins to expand Tregs in individuals with immune-mediated disorders. However, double-blind clinical trials utilizing low-dose IL-2 have produced mixed results. In the LUPIL-2 (NCT02955615), (NCT02465580), and (NCT02932137) studies, primary endpoints were not achieved (55, 56). Similarly, two additional studies with IL-2 muteins (NCT03451422, NCT04433585) did not meet primary endpoints (57, 58). Evidence indicates that lupus is characterized by deficiencies in both IL-2 and TGF-β production (59). If the tolerogenic effects of IL-2 on Treg production require TGF-β (5), then a TGF-β deficiency could contribute to the lack of efficacy observed in lupus clinical trials involving IL-2. Additionally, defects in TGF-β production or signaling have been observed in rheumatoid arthritis, type 1 diabetes, multiple sclerosis, and inflammatory bowel disease (60–62).

Besides the treatment of autoimmune diseases, our NPs should have other clinical indications. Since our NPs can stabilize immune regulation in the tolerogenic state, they may have the capacity to prevent graft rejection. In mice we have shown that we can induce transplantation tolerance without immunosuppression (63). As stated above, they can prevent a lethal graft versus host disease (3).

Moreover, these tolerogenic NPs should be safe and not cause global immunosuppression for four reasons. First, unlike the strong stimulatory effects of anti-CD3, the effects of anti-CD2 coated NPs containing nanomolar amounts of IL-2 on T cells are quite modest (39). Second, broad immunosuppression has not been observed in many clinical trials with exogenous Tregs (64). Third, encapsulation of TGF-β in the NPs will not be necessary. NPs containing IL-2 only were as protective as IL-2 and TGF-β in preventing GVHD. This was because the TGF-β required for Tregs was provided by the tolerogenic NK cells the NPs also induced (5). Thus, the significant toxicity associated with the pleotropic effects of TGF-β should be avoided. Fourth, PLGA nanoparticles are biodegradable and have already been used in clinical trials (65, 66). Biomarkers for NP clinical trials when the translational studies FDA approval are completed will include numbers of Tregs and CD56^bright^ TGF-β^+^ NK cells in PBMC.

In addition to the cellular defects in subjects with autoimmune diseases, Women with recurrent miscarriages may have defects in either decidual Tregs or NK cells (6, 7, 23, 24). Using mouse models to evaluate fetal loss (67), future studies will attempt to use intravaginal CD2 targeted NPs to increase the numbers and function of dTregs and dNK cells at the maternal-fetal interface and decrease fetal loss.

In conclusion, the tolerogenic effects of our CD2-targeted NPs and the prevention of rejection of a semi-allogenic fetus depend upon synergistic TGF-β dependent Tregs and NK cells. The NPs provide IL-2 to the targeted cells *in vivo*, and TGF-β produced by the NK cells they induce supplies the TGF-β essential for the generation and maintenance of Tregs. Correcting deficits in IL-2 and TGF-β through nanoparticle-based approaches may offer potential benefits not only for managing immune-mediated disorders but also recurrent miscarriage. Shifting present approaches from suppressing or depleting immune cells to methods that selectively and safely balance immune homeostasis could become a new paradigm for the treatment of immune-mediated disorders.

## Data Availability

The original contributions presented in the study are included in the article/supplementary material, further inquiries can be directed to the corresponding author/s.
